# The Wilms’ tumor (*WT1*) gene expression correlates with the International Prognostic Scoring System (IPSS) score in patients with myelofibrosis and it is a marker of response to therapy

**DOI:** 10.1002/cam4.735

**Published:** 2016-05-11

**Authors:** Daniela Gallo, Paolo Nicoli, Chiara Calabrese, Valentina Gaidano, Jessica Petiti, Valentina Rosso, Elisabetta Signorino, Sonia Carturan, Giada Bot‐Sartor, Gisella Volpe, Francesco Frassoni, Giuseppe Saglio, Daniela Cilloni

**Affiliations:** ^1^ Department of Clinical and Biological SciencesUniversity of TurinTurinItaly; ^2^ Department of Pediatric Hemato‐Oncology and Stem Cell and Cellular Therapy LaboratoryInstitute G. GasliniLargo G GasliniGenova

**Keywords:** JAK2, molecular marker, myelofibrosis, WT1

## Abstract

The Wilms tumor gene WT1 is a useful marker of clonal hematopoiesis and it has been shown to be a good marker of residual disease and it reflects the response to therapy. Although myelofibrosis is characterized by mutations of JAK2 and calreticulin (CALR), these mutations are not useful to monitor response to therapy. In this study we demonstrated that in patients affected by myelofibrosis WT1 correlates with the International Prognostic Scoring System (IPSS) score at diagnosis. Furthermore WT1 is a good marker of response to JAK2 inhibitors especially for patients without blasts and for patients who develop anemia or thrombocytopenia not for progression but as therapy related toxicity. Finally, WT1 transcript reduction can mirror a benefit of therapy on the disease burden. This study demonstrated that WT1 is a good marker for monitoring the response to therapy in patients affected by myelofibrosis.

Myelofibrosis (MF) is a clonal stem cell disorder characterized by bone marrow fibrosis, extramedullary hematopoiesis, and an increased risk of transformation to acute leukemia. Myelofibrosis may develop as a primary manifestation of MPNs (PMF) or develop from a pre‐existing condition of polycythemia vera (PPV‐MF) or essential thrombocythemia (PET‐MF). A variable degree of leukocytosis and thrombocytosis associated with splenomegaly and progressive bone marrow fibrosis leading to extramedullary hematopoiesis and finally to cytopenias are the main clinical characteristics of primary myelofibrosis.

In 2005, the V617F mutation of Janus kinase 2 (*JAK2*) was discovered 1. The mutation leads to constitutive activation of *JAK2* and contributes to deregulate JAK signaling in myelofibrosis (MF), polycythemia vera (PV), and essential thrombocythemia (ET). This finding generated the illusion of facing a disease with one initiating mutation and possibly a useful drugable target. More recently, two groups identified somatic mutations in exon 9 of calreticulin (*CALR*) encoding the endoplasmic reticulum chaperone calreticulin in about 70–80% of JAK2 unmutated myelofibrosis and ET 2. In addition, the *MPL* mutations are identified in 10% of *JAK2* ^*V617F*^ negative, *CALR* negative myelofibrosis.

The identification of the *JAK2* ^*V617F*^ mutation led to the development of JAK inhibitors, small molecules that selectively target JAK signaling, for the treatment of MPN patients. It was shown in phases II and III clinical trials that ruxolitinib is effective in the reduction of spleen size and disease‐related symptoms. Ruxolitinib is now approved in the United States and Europe for the treatment of myelofibrosis. Several additional compounds are at different stages of clinical development. While most patients with myelofibrosis benefit from ruxolitinib therapy, many of them are resistant or obtain a suboptimal response or loose the response during therapy. Recently, Patel and colleagues demonstrated that the number of mutations may have impact on the response to therapy and finally on overall survival. By contrast, Guglielmelli and colleagues did not find any correlation between mutations and responses. Ruxolitinib has been shown to have limited effect on granulocytic *JAK2* ^*V617F*^ mutant load 3. A modest reduction of the *JAK2* ^*V617F*^ allele burden (8% from baseline at 72 weeks) was observed in MF patients in the COMFORT‐II study. More recently in a phase III study, a reduction in allele burden from baseline was observed and correlated with ruxolitinib treatment response, in particular with reduction in symptoms and spleen volume.

Despite this, the correlation between *JAK2* ^*V617F*^ allele burden and response to treatment is still controversial. Based on the available data, *JAK2* ^*V617F*^ cannot be considered a molecular marker of residual disease or drug response.

In the absence of molecular marker, the response is commonly evaluated based on the IWG‐MRT criteria, recently updated by the European Leukemia Net (ELN) 4. The criteria of complete response include the achievement of a normocellular bone marrow (BM), less than 5% of blasts, and a reduction of fibrosis to less than grade 1. In peripheral blood, the achievement of the complete response is based on the achievement of the Hb level higher than 10 g/dL, neutrophil count higher than 1 × 10^9^/L, and platelets more than 100 × 10^9^/L and less than 2% of immature cells. In addition, reduction in spleen volume and resolution of symptoms are requested to classify the response as complete.

Based on these criteria it is difficult to assess the response to JAK2 inhibitors mainly because of the drug‐related cytopenia. The clinical response and the BM parameters are usually considered to evaluate JAK2 inhibitors response. For patients without an increase of blast cells in the bone marrow and peripheral blood is cumbersome to establish the efficacy of ruxolitinib in controlling the disease. The clinical response and the symptoms assessment are not reliable markers of disease control. Therefore, for many patients affected by primary or secondary myelofibrosis, a molecular marker of response to therapy is still lacking.

The Wilms’ tumor (*WT1*) gene has been demonstrated to be a sensitive molecular marker in acute leukemias, myelodysplastic syndromes, and myeloproliferative disorders. It is now broadly accepted as marker of minimal residual disease after chemotherapy and bone marrow transplantation in acute leukemias 5.

After written informed consent, BM samples were collected from 54 patients affected by myelofibrosis (28 were PMF, 18 PPV‐MF, and 8 PET‐MF). In 32 patients, BM and PB samples are available during follow‐up. A median of five samples are available for each patient, the median time of follow‐up is 28 months (range 12–56).

All the patients were characterized at the molecular level for the presence of *JAK2*,* CALR*, and MPL mutations and for cytogenetic analysis. Of the 54 patients, 32 patients have been treated with ruxolitinib. Ten patients experienced leukemic transformation during follow‐up.

*WT1* has been analyzed in all the patients at diagnosis and during follow‐up as previously described 5. We did not find any significant correlation between *WT1* gene expression and *JAK2* or *CALR* mutations or cytogenetic abnormalities.

As shown in Figure 1A, *WT1* expression levels strictly correlate with the International Prognostic Scoring System (IPSS) at diagnosis. In addition, as already demonstrated in acute leukemias, there is a strict correlation between *WT1* at diagnosis in BM and PB samples (Fig. 1B). The possibility to monitor the disease in PB allows to perform a strict follow‐up and, importantly in myelofibrosis, it allows to overcome the limit of “punctio sicca” which is a common obstacle to disease evaluation.

**Figure 1:**
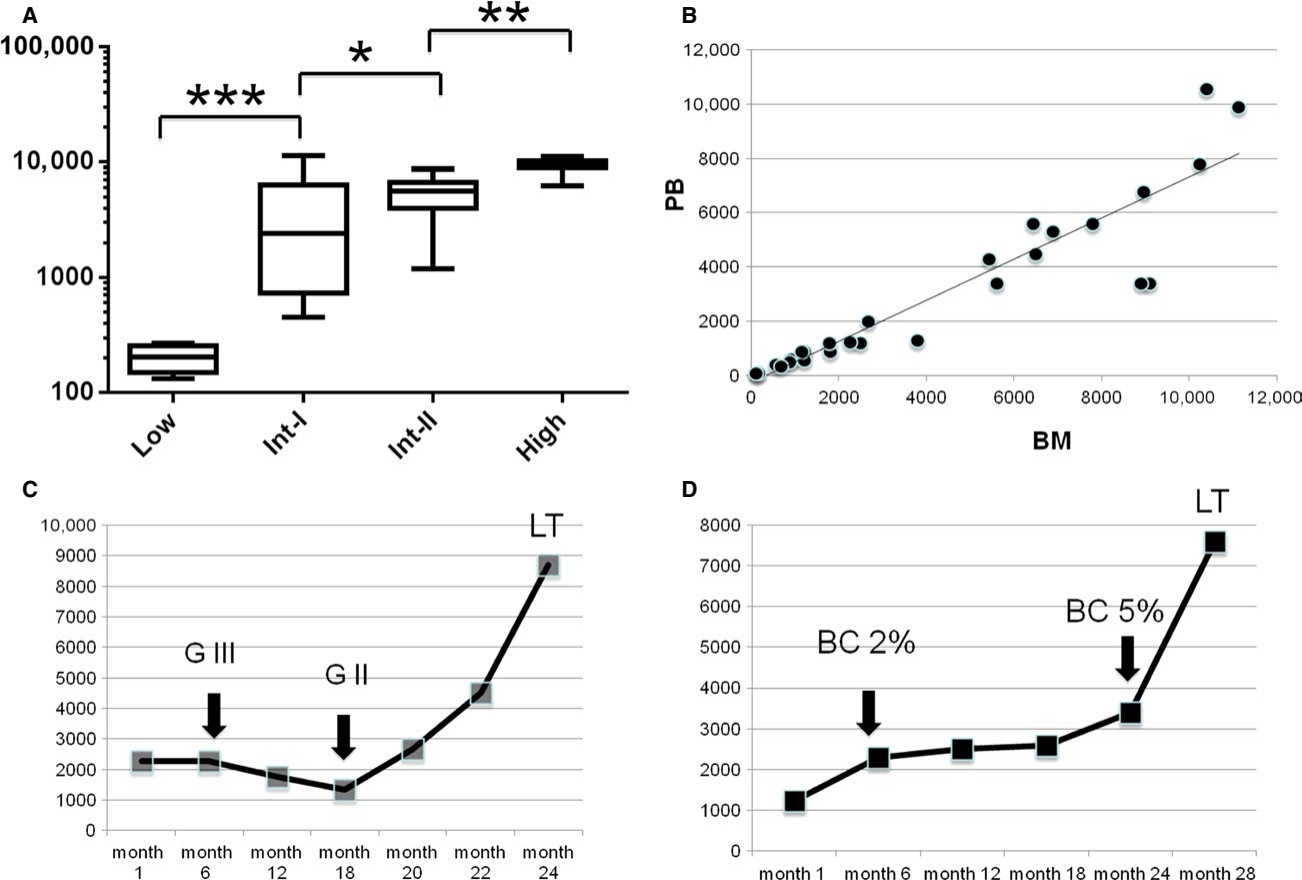
(A) *WT1* expression (*WT1* copies/10^4^ ABL copies) according to the International Prognostic Scoring System (IPSS) risk group. (B) Correlation between *WT1* gene expression in BM and PB (*r* = 0.85). (C) Example of *WT1* expression during follow‐up of a patient treated with ruxolitinib. After 1 year of therapy the patient responded with a reduction of BM fibrosis from grade III (GIII) to grade II (GII). After 20 months of therapy *WT1* started to increase and after 24 months leukemic transformation (LT) was observed. (D) Example of WT1 expression during follow‐up of a patient treated with ruxolitinib. After 6 months of therapy blast cells (BC) were 2%, after 24 months the blast cells were 5%, and *WT1* increased in parallel with the number of blast cells. After 28 months there was a further increase in *WT1* associated with leukemic transformation (LT). * *P* < 0.05; ** *P* < 0.01; *** *P* < 0.001.

In patients who respond to ruxolitinib in terms of reduction in spleen and symptoms, *WT1* progressively decreases, but more importantly, the reduction of *WT1* transcript parallels the reduction of fibrosis and of blast cells (example in Fig. 1C).

To establish the role of *WT1* as marker of disease, we followed patients during disease progression and found a progressive and significant increase of *WT1* that started a median of 3 months before leukemic transformation (range 1–7) when the blood parameters were stable. (Fig. 1C and D). In half of the patients who progressed to leukemia, *WT1* increased several months before when no other signs of transformation were present in bone marrow, in particular no evident increase of blast cells were detected. In those patients, *WT1* was the only parameter which allowed to predict the progression.

To give further strength to these findings, we followed 12 patients who developed significant thrombocytopenia or anemia for drug‐related toxicity, but who did not progress after a median of 3.2 years of follow‐up from the hematological toxicity. In all these patients, *WT1* transcript never increased from baseline.

Based on these data, *WT1* is a useful marker of disease and of response to JAK2 inhibitors especially for patients without blasts and for patients who develop anemia or thrombocytopenia not for progression but as therapy‐related toxicity. Finally, *WT1* transcript reduction can mirror a benefit of therapy on the disease burden even in the absence of spleen reduction or of improvement of symptoms score. Finally, the possibility to monitor the disease even in PB offers the opportunity to perform a strict follow‐up with a better compliance of both, patients and clinicians, and to overcome the obstacle due to the failure of the bone marrow aspirate in patients affected by myelofibrosis.

## Conflict of Interest

None declared.
